# Characterization of group A streptococci causing invasive diseases in Sri Lanka

**DOI:** 10.1099/acmi.0.000697.v4

**Published:** 2024-06-06

**Authors:** Madumali Weerasekara, Gihani Vidanapathirana, Carmen Li, Asanka Tennegedara, Rasadanie Dissanayake, Asela Ekanayake, Muditha Abeykoon, Mahen Kothalawala, Veranja Liyanapathirana, Margaret Ip

**Affiliations:** 1 Department of Medical Laboratory Science, Faculty of Allied Health Sciences, University of Peradeniya, Peradeniya, Sri Lanka; 2 Department of Microbiology, Faculty of Medicine, University of Peradeniya, Peradeniya, Sri Lanka; 3 Department of Microbiology, Faculty of Medicine, The Chinese University of Hong Kong, Hong Kong, SAR; 4 National Hospital, Kandy, Sri Lanka; 5 National Hospital of Sri Lanka, Colombo, Sri Lanka

**Keywords:** antibiotic resistance, *emm* typing, group A streptococci, virulence markers

## Abstract

Group A β haemolytic streptococcus (GAS) or *Streptococcus pyogenes* is a human pathogen that causes an array of infections, including pharyngitis, cellulitis, impetigo, scarlet fever, toxic shock syndrome, and necrotizing fasciitis. The present study characterizes 51 GAS isolates from invasive infections in Sri Lanka, focusing on resistance profiles, genetic determinants of resistance, and virulence markers. Isolates were tested for sensitivity to penicillin, erythromycin, clindamycin, and tetracycline. The presence of *erm*(A), *erm*(B), and *mef*(A) was detected in erythromycin-resistant isolates, while *tet*(M) was detected in the tetracycline-resistant isolates. PCR was used to identify SpeA, SpeB, SpeC, SpeF, SpeG, *smez*, and *ssa* as virulence markers. Selected GAS isolates were *emm*-typed using the updated CDC protocol. All 51 isolates were susceptible to penicillin. The number of isolates non-susceptible to erythromycin was 16. The commonest resistance determinant identified was *erm*(B) (11/16). Tetracycline non-susceptibility was found in 36 (70.6 %) isolates and 26 of them contained the *tet*(M) gene. Thirteen (25.5 %) isolates were resistant to both tetracycline and erythromycin, while 12 (23.5 %) isolates were sensitive to both antibiotics. The commonest virulence markers detected among the isolates were SpeB (44, 86.3 %), SpeG (36, 70.6 %), and SpeF (35, 68.6 %), while SpeJ (15, 29.4 %), SpeA (10, 19.6 %), and *ssa* (5,9.8 %) were less common. The *emm* types were diverse. In conclusion, the GAS isolates studied showed resistance to erythromycin and tetracycline, while retaining universal susceptibility to penicillin. Additionally, these isolates exhibited diverse genetic backgrounds, displaying varying patterns of virulence genes and *emm* types.

## Data Summary

Supplementary database is avaliable with the online version of this article. All sequences have been deposited in GenBank with accession numbers: OR463405, OR463406, OR463407, OR463408, OR463409, OR463410, OR463411, OR463412, OR463413, OR463414, OR463415, OR463416, OR463417, OR463418, OR463419.

## Introduction

Group A β haemolytic streptococcus (GAS) or *Streptococcus pyogenes* is a successful human pathogen. Despite being identified as a human pathogen for many years, many aspects of the bacteria and the disease burden remains to be studied globally, particularly in the Global South.

Conditions such as improved quality of living ha led to the reduction of some GAS-related diseases, such as rheumatic heart diseases. However, there have been increasing reports of invasive GAS (iGAS) diseases [1].

The pathogenicity of GAS is dependent on their virulence markers. M proteins are a major virulence determinant of GAS. Other recognized virulence markers include GAS superantigens (SAgs). There are different types of SAgs in GAS. These include streptococcal pyrogenic exotoxins (SPEs), streptococcal mitogenic exotoxins (SMEs), and streptococcal superantigens (SSAs). Some of these are chromosomally encoded, while others are associated with bacteriophages [2]. Their distribution has been identified to be varied [3]. Other virulence factors include opacity factor, streptolysin O and F, C5a protease, hyaluronidase, and streptokinase [2].

GAS have different subtypes. Traditionally, they have been subtyped based on the M protein, which is also an important virulence marker. Over 50 different serotypes were first identified using the M typing method [4]. However, the typing of GAS isolates has now changed based on the advent of molecular techniques. The most commonly implemented method is *emm* typing. The *emm* gene encodes for the M protein and sequences of its 5′ end have been used globally to type GAS isolates [5]. So far, more than 200 *emm* types and more than 750 sub-types have been identified. The predominant types are shown to vary across different regions of the world [5]. However, uncommon types have been shown to increase globally in different parts of the world [6]. There is also evidence of persistence and expansion of clones associated with antibiotic resistance [7,8]. In some countries, specific *emm* types are associated with particular disease types [9]. However, there is also evidence that many *emm* types could be responsible for the same disease entity [10]. Thus, there is a need to understand more about the pathogenicity of different *emm* types in different locales.

In Sri Lanka too, there is an apparent increase in iGAS diseases (personal communications with clinical microbiologists). A literature search with the keywords group A streptococcus, *Streptococcus pyogenes* and Sri Lanka fails to identify any literature describing the epidemiology or biology of GAS within Sri Lanka. Further, *emm* subtypes from Sri Lanka are not available within the *emm* databases to the best of our knowledge. Given that *emm* type-based, M protein-targeting vaccines are being developed against GAS, identifying the types present within a country, particularly those responsible for invasive diseases, is important.

This study aimed to characterize GAS isolates obtained from patients with invasive diseases from selected sites in Sri Lanka by identifying their resistance profiles, genetic markers of resistance, and selected virulence markers. Further, typing of selected isolates was also performed through *emm* typing.

## Methodology

### Isolates

Fifty-one isolates of GAS collected from the National Hospital, Kandy, Provincial General Hospital, Polonnaruwa, Teaching Hospital, Peradeniya, and District General Hospital, Kegalle, Sri Lanka were characterized for this study (Table 1), after obtaining ethical approval from the Ethics Review Committee, Faculty of Allied Health, Sciences, University of Peradeniya, Sri Lanka (AHS/ERC/2021/101). Isolates were obtained from blood cultures or deep tissue samples. In the case of deep tissue samples, they were obtained from patients with a diagnosis of necrotizing fasciitis or any other clinically diagnosed invasive skin and soft tissue infection. Data related to the isolates were obtained through the request forms forwarded by the co-investigators at the respective hospitals.

**Table 1.** Year and province of isolation

Characteristic	No. (%)
Year of isolation	
2014	1 (2 %)
2015	1 (2 %)
2016	1 (2 %)
2017	17 (33.3 %)
2018	2 (3.9 %)
2020	29 (56.9 %)
Province of origin	
Central Province Sri Lanka	19 (27.3 %)
North-Central Province Sri Lanka	15 (29.4 %)
Sabaragamuwa Province, Sri Lanka	12 (23.5 %)
Unknown	5 (9.8 %)

### Laboratory procedure

All isolates were taken from storage at – 80 °C and passaged twice on blood agar before further analysis was performed. The isolates were identified as GAS via bacitracin susceptibility testing as well as Lancefield grouping with latex agglutination (Oxoid, UK).

### Antimicrobial susceptibility testing

All isolates were tested for sensitivity to penicillin (10 U), erythromycin (15 µg), clindamycin (2 µg), and tetracycline (30 µg) with disc diffusion testing [11].

### Identification of virulence markers

SpeA, SpeB, SpeC, SpeF, SpeG, *smez*, and *ssa* genes were identified through previously described PCRs [3,12]

### Identification of resistance markers

The presence of *erm*(A), *erm*(B), and *mef*(A) was identified in erythromycin-resistant isolates, while *tet*(M) was identified in the tetracycline-resistant isolates using previously described protocols [13].

### Molecular epidemiology

Isolates from 2017 were *emm*-typed using the updated CDC protocol [14].

### Data analysis

Data were entered into an MS Excel database and analysed via SPSS (version 21) (Supplementary File 1). Percentages were calculated for the year of isolation, origin, antibiotic susceptibility patterns, and presence of genetic markers and virulence factors. The potential association between resistance to tetracycline and erythromycin was assessed using a Chi-square test for association.

## Results and discussion

### Antibiotic susceptibility patterns and resistant determinants

All 51 isolates were susceptible to penicillin, which is in agreement with the well-established norm [15].

Four (7.8 %) isolates were resistant to clindamycin. Erythromycin non-susceptibility (either intermediately sensitive or resistant) was seen in 16 (31.4 %) isolates and tetracycline non-susceptibility was found in 36 (70.6 %) isolates (Table 2).

**Table 2.** Antibiotic sensitivity patterns of the study isolates

Antibiotics	Sensitive*n* (%)	Intermediate sensitive*n* (%)	Resistant*n* (%)
Penicillin 10 U	51 (100 %)	0 (0 %)	0 (0 %)
Tetracycline 30 µg	15 (29.4 %)	2 (3.9 %)	34 (66.7 %)
Clindamycin 2 µg	47 (92.2 %)	2 (3.9 %)	2 (3.9 %)
Erythromycin 15 µg	35 (68.6 %)	0 (0 %)	16 (31.4 %)

Thirteen (25.5 %) isolates were non-susceptible to both tetracycline and erythromycin, while 12 (23.5 %) isolates were sensitive to both antibiotics. Three isolates (5.9 %) were resistant to erythromycin alone, while 23 (45.1 %) were resistant to tetracycline alone. In contrast to penicillin susceptibility, resistance to other classes of antimicrobial drugs has shown a gradually increasing trend among GAS isolates globally [16]. Over the past few decades, increasing macrolide resistance of GAS has been reported. Globally, resistance rates have been higher in some countries, such as PR China [17] and India [18], while it has been lower in some other countries, such as Japan and Taiwan, ROC [19].

Of the 16 erythromycin non-susceptible isolates, 11 had the *erm*(B) gene, 3 had the *mef*(A) gene, and 1 isolate had the *erm*(A) gene. Of the 36 isolates non-susceptible to tetracycline, 26 contained the *tet*(M) gene. Co-occurrence patterns for the resistant determinants are given in Table 3. *Erm*(B) has been identified as the commonest resistant determinant for erythromycin resistance in many studies [17,18, 20].

**Table 3.** Co-occurrence patterns for the resistant determinants

Resistant genes	*n*
*erm*(A) alone	0
*erm*(B) alone	4
*mef*(A) alone	1
*tet*(M) alone	17
*tet*(M)+*erm*(A)	1
*tet*(M)+*erm*(B)	6
*tet*(M)+*mef*(A)	1
*tet*(M)+*erm*(B)+*mef*(A)	1
None*	20
Total	51

* Of these, 12 were sensitive to both antibiotics, 7 were resistant to tetracycline, and one1 was resistant to both antibiotics. Percentages were not calculated as only the nonsusceptible isolates were tested.

A recent study conducted in Central Africa reported 80 % tetracycline resistance among 76 GAS clinical isolates, which is parallel to the findings of our study [21]. Ayer *et al*. identified 90 % (*n*=72) of the GAS isolates with *tet*(M) gene [22]. Further, they determined the relationship between resistance to tetracycline and macrolides in GAS. According to their findings, the *tet*(M) gene was found in association with *erm*(B) in 71 % of the total *erm*(B)-positive isolates. Similarly, we identified that 7 of the 13 isolates that were non-susceptible for both erythromycin and tetracycline were positive for both *erm*(B) and *tet*(M).

Resistance to tetracycline is naturally conferred by ribosome protection genes, such as *tet*(M) and *tet*(O) in Gram-positive bacteria. Tetracycline resistance genes can be located on mobile genetic elements that carry macrolide resistance genes, which enable the co-occurrence of resistance to both classes of antibiotics [23]. This is the likely reason for the concurrent occurrence of the two genes.

Considering the antibiotic susceptibility patterns, while universal susceptibility to penicillin is encouraging, the higher rates of resistance to tetracycline and erythromycin is a concern that the wider clinical community should be aware of, since erythromycin is a commonly used alternative for GAS infections in the presence of penicillin allergy.

### Virulence markers

**Fig. 1.**
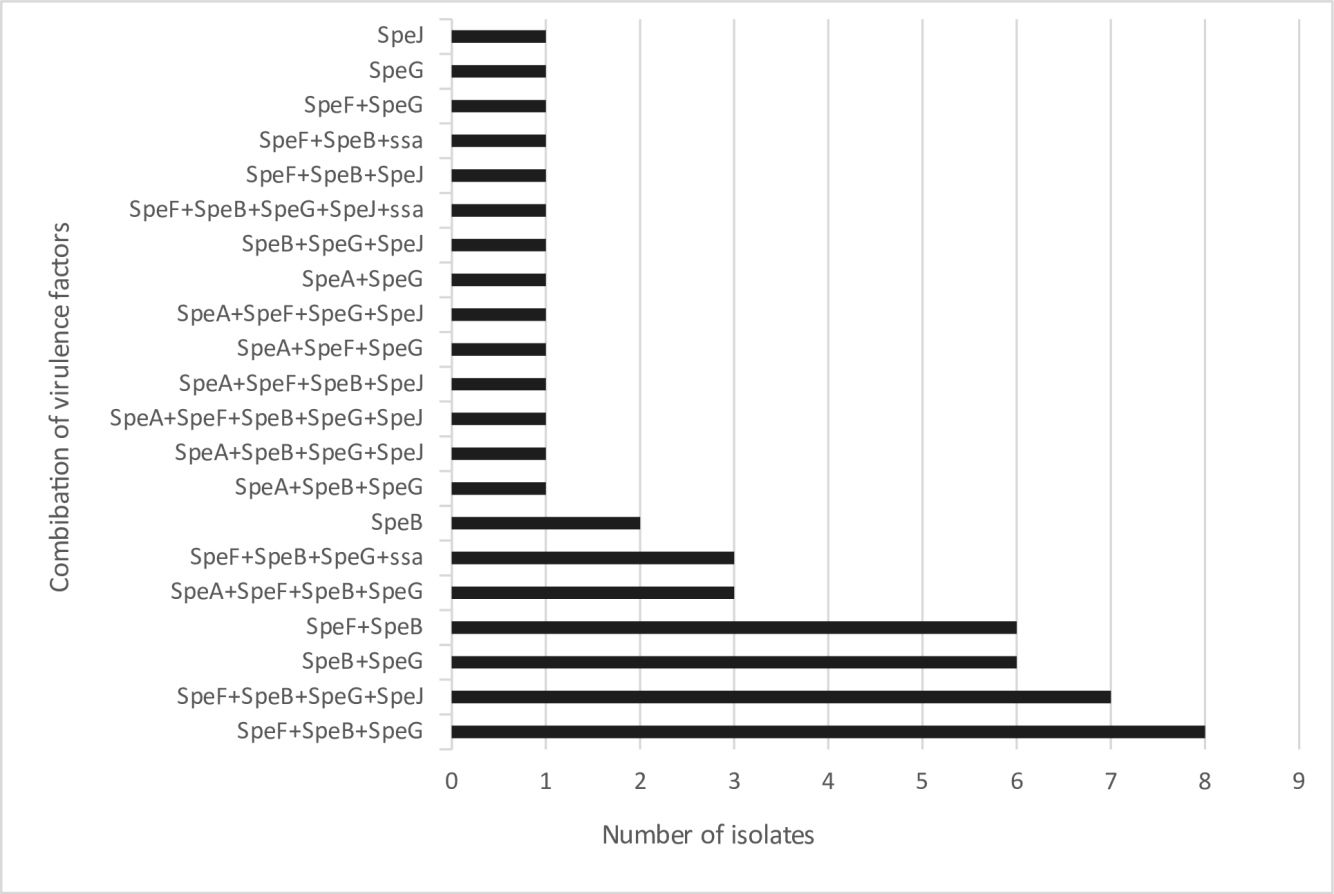
Co-occurrence pattern of virulence determinants (two isolates did not contain any of the selected virulent factors).

The commonest virulence markers detected among the isolates were SpeB (44, 86.3 %), SpeG (36, 70.6 %), and SpeF (35, 68.6 %), while SpeJ (15, 29.4 %), SpeA (10, 19.6 %), and *ssa* (5,9.8 %) were less common. The co-occurrence pattern of the virulence determinants is given in Fig. 1.

In other countries too, SpeB, SpeG, and SpeF have been found to be commonest virulence determinants [24]. Streptococcal pyrogenic exotoxin B (SpeB) has been identified to be the commonest secreted protein present among a majority of clinically identified GAS isolates. Its function is a subject of ongoing debate, as contradictory findings have been identified by different groups [25]. However, there are more than sufficient data currently to suggest that it plays an important contributory role in causing infections [26]. Therefore, identifying it as the commonest virulence marker is expected. Streptococcal pyrogenic exotoxin G (SpeG) is chromosomally encoded but is not found in all isolates [27]. However, many studies have identified it to be present in a majority of isolates [24,27]. SpeF has also been found in higher concentrations among antibiotic-resistant GAS isolates in India [28]. Superantigens (SpeA, SpeC, SpeH, SpeJ, SpeZ, *smez*) are associated with invasiveness in different studies [29,30].

Research evidence supports that the phage-encoded speA and speC genes are associated with invasive infection. A high rate of speA and speC gene carriage has frequently been identified among streptococcal toxic shock syndrome-associated isolates compared with those isolated from superficial infections [29,30].

An Italian study that characterized GAS clinical isolates reported SpeB as the commonest virulence gene, which is in agreement with the present study. SpeC, SpeH, and *smez* were also identified among their isolates [31]. However, a Finish study aiming to identify superantigen profiles of GAS isolates from patients with bacteraemia has identified SpeA and SpeC in 20 and 30 % of the strains, respectively, as the commonest virulence determinants [29]. These were less prevalent in our study. In a recent study by Lintges *et al*., superantigens were found to be more important for the invasiveness of GAS, with SpeA, SpeJ, and SpeZ having the greatest invasive potential [32].

*Ssa* is a phage-encoded superantigen that may co-occur with SpeA. In recent studies, *ssa* has been associated with scarlet fever, while it has also been found in association with other isolates [17,33]. Our isolate cohort showed a lower prevalence of both *ssa* and SpeA. Our isolates were from invasive diseases that are mostly of musculoskeletal origin rather than scarlet fever. This could be a reason for the low prevalence of *ssa* and SpeA.

The overall patterns of virulence factors are in keeping with the clinical picture patients from whom the isolates were obtained. However, to understand the action and clinical significance of these virulence factors fully, a wider study, including both invasive and non-invasive isolates as well as a carriage component, is warranted.

### *emm* types

Diverse group of *emm* types were identified for the 15 isolates that were typed (Table 4).

**Table 4.** *emm* types identified

Sample	Year isolation	Place of isolation	*emm* type
STRE14	2017	North Central	92.0
STRE15	2017	North Central	77
STRE16	2017	North Central	93.7
STRE17	2017	North Central	92.7
STRE18	2017	North Central	28.5
STRE20	2017	North Central	68
STRE21	2017	North Central	68
STRE22	2017	North Central	81.1
STRE23	2017	North Central	232.1
STRE24	2017	North Central	232
STRE25	2017	North Central	105
STRE26	2017	North Central	9
STRE27	2017	North Central	131
STRE31	2017	Sabaragamuwa	9
STRE32	2017	Sabaragamuwa	9

The M protein gene (*emm*) encodes the cell surface M virulence protein responsible for at least 100 *Streptococcus pyogenes* M serotypes. While we could not perform *emm* typing on all isolates, *emm* types 9, 92, 68, and 232 were identified in more than one isolate. A systematic review that focused on global *emm* type distribution of group A streptococci has reported that the *emm* typing related epidemiological data from high-income countries were predominant while data from low-income countries were deficient [5]. Further, a significant diversity can be identified among the *emm* types across different geographical areas [34].

### Limitations

This is the first study in Sri Lanka to characterize the GAS causing invasive diseases with profiling of genetic determinants of resistance, virulence genes, and *emm* types. However, there are some limitations in the present study. The availability of partial funding led us to identify a limited number of resistant determinants, virulence markers (six), and limited *emm* typing, which does not describe the full picture of the isolates present. We lack clinical data for a meaningful analysis of associations with clinical outcomes. While the lack of clinical data is a major drawback, since this is the first time that GAS isolates are being described from Sri Lanka, we consider that the higher antibiotic resistance rates, higher presence of resistance markers, and the presence of diverse *emm* types indicate a broad spread of genetically diverse group A streptococci in the country, which could pose a treatment challenge and warrants regular surveillance. As highlighted by Steer *et al*. in their systematic review [5], there is a lack of global epidemiological data on GAS from developing countries. Therefore, despite being a small study, our data add value to the global literature.

## Conclusion

We have demonstrated that while the universal susceptibility to penicillin remains, resistance to erythromycin and tetracycline is prevalent in GAS isolates in Sri Lanka. Further, these isolates are from diverse backgrounds with different patterns of virulence genes and *emm* types. This preliminary study indicates that there is a necessity to monitor invasive bacterial isolates such as GAS globally, particularly from countries with poor data generation, as this would benefit the background work in the search for potential vaccine types and in the treatment of patients.

## supplementary material

10.1099/acmi.0.000697.v4 Supplementary Material 1.

## Abbreviations

GAS: group a streptococcus
iGAS: invasive group a streptococcus
sAgs: superantigens
SMEs: streptococcal mitogenic exotoxins
SPEs: streptococcal pyrogenic exotoxins
SSAs: streptococcal superantigens

## References

[1] Efstratiou A, Lamagni T (2022). Streptococcus pyogenes: Basic Biology to Clinical Manifestations.

[2] Jasim SA, Hatem ZA, Abd Mohammed Z (2021). Annals of the Romanian Society for Cell Biology.

[3] Commons R, Rogers S, Gooding T, Danchin M, Carapetis J (2008). Superantigen genes in group A streptococcal isolates and their relationship with emm types. J Med Microbiol.

[4] Vekemans J, Gouvea-Reis F, Kim JH, Excler J-L, Smeesters PR (2019). The path to group A *Streptococcus* vaccines: World Health Organization Research and Development Technology roadmap and preferred product characteristics. Clin Infect Dis.

[5] Steer AC, Law I, Matatolu L, Beall BW, Carapetis JR (2009). Global emm type distribution of group A streptococci: systematic review and implications for vaccine development. Lancet Infect Dis.

[6] Chiang-Ni C, Wu A-B, Liu C-C, Chen K-T, Lin Y-S (2011). Emergence of uncommon emm types of *Streptococcus pyogenes* among adult patients in southern Taiwan. J Microbiol Immunol Infect.

[7] Rafei R, Hawli M, Osman M, Dabboussi F, Hamze M (2020). Distribution of emm types and macrolide resistance determinants among group A streptococci in the Middle East and North Africa region. J Glob Antimicrob Resist.

[8] Tsai W-C, Shen C-F, Lin Y-L, Shen F-C, Tsai P-J (2021). Emergence of macrolide-resistant *Streptococcus pyogenes* emm12 in southern Taiwan from 2000 to 2019. J Microbiol Immunol Infect.

[9] Gröndahl-Yli-Hannuksela K, Beres SB, Hyyryläinen HL, Kallonen T, Musser JM (2021). Genetic evolution of invasive emm28 *Streptococcus pyogenes* strains and significant association with puerperal infections in young women in Finland. Clin Microbiol Infect.

[10] de Crombrugghe G, Baroux N, Botteaux A, Moreland NJ, Williamson DA (2020). The limitations of the rheumatogenic concept for group A *Streptococcus*: systematic review and genetic analysis. Clin Infect Dis.

[11] Clinical and laboratory standards institute (2020). Performance Standards for Antimicrobial Susceptibility Testing.

[12] Khan RMA, Anwar S, Pirzada ZA (2020). *Streptococcus pyogenes* strains associated with invasive and non-invasive infections present possible links with *emm* types and superantigens. Iran J Basic Med Sci.

[13] Rivera A, Rebollo M, Miró E, Mateo M, Navarro F (2006). Superantigen gene profile, emm type and antibiotic resistance genes among group A streptococcal isolates from Barcelona, Spain. J Med Microbiol.

[14] Frost HR, Davies MR, Velusamy S, Delforge V, Erhart A (2020). Updated emm-typing protocol for *Streptococcus pyogenes*. Clin Microbiol Infect.

[15] Stevens DL, Bisno AL, Chambers HF, Everett ED, Dellinger P (2005). Practice guidelines for the diagnosis and management of skin and soft-tissue infections. Clin Infect Dis.

[16] Silva-Costa C, Friães A, Ramirez M, Melo-Cristino J (2015). Macrolide-resistant *Streptococcus pyogenes*: prevalence and treatment strategies. Expert Rev Anti Infect Ther.

[17] Li H, Zhou L, Zhao Y, Ma L, Zhang H (2023). Epidemiological analysis of group A streptococcus infection diseases among children in Beijing, China under COVID-19 pandemic. BMC Pediatr.

[18] Abraham T, Sistla S (2018). Trends in antimicrobial resistance patterns of group A streptococci, molecular basis and implications. Indian J Med Microbiol.

[19] Arêas GP, Schuab RBB, Neves FPG, Barros RR (2014). Antimicrobial susceptibility patterns, emm type distribution and genetic diversity of *Streptococcus pyogenes* recovered in Brazil. Mem Inst Oswaldo Cruz.

[20] Liu X, Shen X, Chang H, Huang G, Fu Z (2009). High macrolide resistance in *Streptococcus pyogenes* strains isolated from children with pharyngitis in China. Pediatr Pulmonol.

[21] Arnold B, Bélard S, Alabi A, Hufnagel M, Berner R (2022). High diversity of emm types and marked tetracycline resistance of group A streptococci and Other ß-hemolytic Streptococci in Gabon, Central Africa. Pediatr Infect Dis J.

[22] Ayer V, Tewodros W, Manoharan A, Skariah S, Luo F (2007). Tetracycline resistance in group A Streptococci: emergence on a global scale and influence on multiple-drug resistance. Antimicrob Agents Chemother.

[23] Giovanetti E, Brenciani A, Lupidi R, Roberts MC, Varaldo PE (2003). Presence of the tet(O) gene in erythromycin- and tetracycline-resistant strains of *Streptococcus pyogenes* and linkage with either the mef(A) or the erm(A) gene. Antimicrob Agents Chemother.

[24] Golińska E, van der Linden M, Więcek G, Mikołajczyk D, Machul A (2016). Virulence factors of *Streptococcus pyogenes* strains from women in peri-labor with invasive infections. Eur J Clin Microbiol Infect Dis.

[25] Nelson DC, Garbe J, Collin M (2011). Cysteine proteinase SpeB from *Streptococcus pyogenes* – a potent modifier of immunologically important host and bacterial proteins. bchm.

[26] Olsen RJ, Raghuram A, Cantu C, Hartman MH, Jimenez FE (2015). The majority of 9,729 group A streptococcus strains causing disease secrete SpeB cysteine protease: pathogenesis implications. Infect Immun.

[27] Reglinski M, Sriskandan S, Turner CE (2019). Identification of two new core chromosome-encoded superantigens in *Streptococcus pyogenes*; speQ and speR. J Infect.

[28] Ray D, Saha S, Sinha S, Pal NK, Bhattacharya B (2016). Molecular characterization and evaluation of the emerging antibiotic-resistant *Streptococcus pyogenes* from eastern India. BMC Infect Dis.

[29] Rantala S, Vähäkuopus S, Siljander T, Vuopio J, Huhtala H (2012). *Streptococcus pyogenes* bacteraemia, emm types and superantigen profiles. Eur J Clin Microbiol Infect Dis.

[30] Maripuu L, Eriksson A, Norgren M (2008). Superantigen gene profile diversity among clinical group A streptococcal isolates. FEMS Immunol Med Microbiol.

[31] Bencardino D, Di Luca MC, Petrelli D, Prenna M, Vitali LA (2019). High virulence gene diversity in *Streptococcus pyogenes* isolated in Central Italy. PeerJ.

[32] Lintges M, van der Linden M, Hilgers R-D, Arlt S, Al-Lahham A (2010). Superantigen genes are more important than the emm type for the invasiveness of group A Streptococcus infection. J Infect Dis.

[33] Hurst JR, Brouwer S, Walker MJ, McCormick JK (2021). Streptococcal superantigens and the return of scarlet fever. PLoS Pathog.

[34] McMillan DJ, Drèze P-A, Vu T, Bessen DE, Guglielmini J (2013). Updated model of group A *Streptococcus* M proteins based on a comprehensive worldwide study. Clin Microbiol Infect.

